# Supplementary material for: I Told You So! Verbal cue beliefs are associated with truth detection, but not lie detection
Source: PLoS One. 2026 Jan 23;21(1):e0339531. doi: 10.1371/journal.pone.0339531 (PMC12829793; doi:10.1371/journal.pone.0339531)
Supplement: S1 Appendix — (DOCX) [file pone.0339531.s001.docx]

# **Appendix** **A**

1. Coherence (CBCA)
   The extent to which all elements of a statement are logically connected and form a unified whole.
2. Clarity (RM)
   The degree to which a statement is clear, sharp, and vivid (instead of dim and vague).
3. Spontaneous Corrections (CBCA)
   Refers to corrections that are made or information that is added to material previously provided in the statement without having been prompted by the interviewer. For example: ‘It was already 10 o’clock, oh no wait 11 o’clock’.
4. Contradictions (CBCA)
   The extent to which a statement contains elements that contradict each other. For example, at the beginning of a statement someone reported that the perpetrator entered the house around midnight, while saying later that the perpetrator broke in at 2.00 AM.
5. Perceptual Information (RM)
   Refers to the presences of sensory information in a statement. Does the statement include sensorial experiences such as sounds (‘He shouted at me’), smells (‘it had a smell of rotten fish’), physical sensations (‘It really hurt’), and visual details (‘I saw him entering the house’).
6. Described Emotions (CBCA, RM)
   Refers to information that describes how the person felt during an event and how these feelings develop and change throughout an event (‘I was very scared, but relieved when it was all over’).
7. Quantity of Details (CBCA)
   Refers to whether a statement is rich in detail and includes specific descriptions of place, time, persons, objects, and events.
8. Spatial Details (CBCA, RM)
   Refers to information about locations (‘It happened in a park’) or the spatial arrangement of people and/or objects (‘The man was sitting to the left of his wife’).
9. Unstructured Production (CBCA)
   Refers to the chronological order in which the statement is told. Are they told in the same order in which they occurred (chronological), or not (unstructured)?
10. Interactions (CBCA)
    Refers to how action and reaction of the different actors presented in the statement are linked. For example, ‘I said go away but he didn’t, he just smiled, and then I started crying’.
11. Temporal Details (CBCA, RM)
    Refers to information about when the event happened (‘It was early in the morning’, ‘an hour after I left’) or explicitly describes a sequence of events (‘When the visitor heard all that noise, he became nervous and left’).
12. Superfluous Details (CBCA)
    Refers to irrelevant details that are related to the event, but not necessary to understand the event. For example, a witness who says that the perpetrator tried to get rid of the cat that entered the bedroom because the perpetrator is allergic to cats.
13. Reproduction of Conversation (CBCA)
    Refers to whether parts of the conversation are reported in original form or if the different speakers are recognizable in the reproduced dialogs. For example, he asked: ‘Are you okay?’
14. Reconstructability (RM)
    Refers to whether it is possible to reconstruct the event on the basis of the information given.
15. Unusual Details (CBCA)
    Refers to details of people, objects, or events that are unique, unexpected, or surprising but meaningful in the context. Examples are a witness who gives a description of a tattoo on the perpetrator’s arm, or a witness who says that the perpetrator had a stutter.
16. Plausibility (RM)
    Refers to whether the story is plausible and realistic and makes sense.
17. Cognitive Operations (RM)
    These cue descriptions of inferences are made by the participant based on existing knowledge. For example, ‘It must have been cold because I had my winter coat’ or ‘It seemed to me that he/she did not recognize this building’ is a conclusion based on observed behavior.
