# Supplementary material for: I Told You So! Verbal cue beliefs are associated with truth detection, but not lie detection
Source: PLoS One. 2026 Jan 23;21(1):e0339531. doi: 10.1371/journal.pone.0339531 (PMC12829793; doi:10.1371/journal.pone.0339531)
Supplement: Attachment — Submitted filename: Response to reviewers.docx [file pone.0339531.s003.docx]

Dear Dr. Wojciechowski,

We would like to thank you and the reviewers for the thoughtful feedback on our manuscript entitled “I Told You So! Verbal Cue Beliefs Are Associated with Truth Detection, but Not Lie Detection”. We believe the comments helped us to substantially improve the manuscript.

We have carefully addressed all points raised, including those mentioned in your summary, and respond to them in detail in the point-by-point replies below. Where relevant, we indicate how and where changes have been implemented in the revised manuscript. For your convenience, we also included a version of our manuscript with tracked changes.

With kind regards,

The authors

**Comments to the Author**

**Reviewer #1:**

The study addresses the vital question of whether knowledge about valid verbal cues can improve truth/lie discrimination by attempting to replicate and extend previous findings on this relationship. The manuscript is well written, clear, and engaging. The authors present their ideas in a precise and well-structured manner. The argumentation is coherent and it is concise and to the point — it conveys all the necessary information without being overly wordy or redundant. I also greatly appreciate the strong methodological rigor demonstrated in this manuscript. The authors should be commended for their careful approach to study design, including the use of formal sample size estimation, very precise operationalization of the variables, pretesting video recordings and good and clear presentation of results. Of course ideally all of this should be considered a baseline standard for research in the detection deception area. Unfortunately, I have seen many studies with considerably weaker methodological practices, so this level of rigor is both commendable and refreshing.

**Thank you for this compliment.**

1. I was a little surprised by the lack of modality effect on truth/lie discrimination accuracy or credibility judgments. I believe that it might be - as Authors wrote, due to stimuli. And stimuli is my main and only concern of this study. The stimuli utilized in this study were notably brief. Specifically, the mean duration of the video statements was 95.39 seconds, which is roughly 1.5 minutes. This restricted duration, coupled with the structured nature of the autobiographical statements, suggests that the viewing time might have been insufficient for many non-diagnostic visual distractors - such as nervous behaviors often stereotypically associated with lying - to fully emerge or influence judgment consistently. As a result, this brevity may have prevented a reduction in deception detection accuracy in the visual modality compared to the transcript-based condition. This suggests that the study has rather limited applied value (for example, for forensic or investigative contexts), although it remains valuable as a careful replication of the established relationship between beliefs about deception cues and detection accuracy.

**As suggested, we addressed this issue in our limitations: “*Second, the deception stimuli used consisted of brief, structured autobiographical statements. While this design ensured control and comparability, it may not fully capture the complexities of deception in real world deception detection contexts. Furthermore, the short duration may have reduced the potential for visual cues to act as (non-diagnostic) distractors, potentially explaining the lack of modality effects. As such, caution is warranted when generalizing these findings to applied settings.*”**

2. While the preregistration and the final manuscript are highly consistent, there were very small and justified differences that reflect a refinement in the reporting of the research. I appreciate the authors’ explanation of those differences as this point initially caught my attention and was my point of concern. Their clarification demonstrates transparency and strengthens the credibility of the research process. I strongly advise reviewing the final text once more, specifically focusing on consistency in terminology and minor reporting details.
   a. For example Authors In the Results section, when describing the ANOVA analysis state that a 3 (Modality: Audio, Visual, Audiovisual) × 2 (Veracity: Truth vs. Lie) analysis was conducted. However in all manuscript they described Audio, Transcript, and Audiovisual design.

**We thank the reviewer for pointing this out. We have carefully reviewed the manuscript and revised all terminology to ensure consistency. Specifically, we now consistently refer to the three conditions as audio-only, transcript, and audio-visual throughout the manuscript**

b. There are also several other minor errors of this kind, which, although small, make the text more difficult to read and follow.

**We thoroughly reviewed the ms and hope to have corrected all minor errors.**

Overall, the manuscript is very well written and methodologically sound. It represents a solid and careful piece of work, and the replication is executed with commendable rigor. That being said, in my opinion, while the study is of high quality, it does not contribute substantially new insights to the psychology of deception. In my view, the paper is a valuable replication study with a little “twist” that strengthens the empirical foundation of the field, but it is unlikely to change current theoretical perspectives or significantly advance the discipline.
Overall, I recommend the manuscript for publication, provided that the journal is open to publishing strong replications, even if they do not offer major conceptual innovation.

**Thank you.**

**Reviewer #2:**

The findings of this study are not particularly surprising. They replicate Bogaard and Meijer’s finding with their results on their binary truth/lie judgment and find a similar lack of effect due to statement modality seen frequently in the literature. I defer to the action editor to decide if that is novel enough to warrant publication.

**We appreciate the reviewer’s comment and take it as a compliment to the quality of the original study. While we understand that the replication of Bogaard and Meijer’s findings may not be seen as particularly surprising, we respectfully note that successful replications, especially those that closely mirror original procedures and still yield consistent outcomes, are far from trivial.**

Other than that, I found the study to be soundly conducted and the results and conclusions reasonable. There are several things that would strengthen this article:

1. Please state your design early in the method. The authors mention the 2x3 but please specify exactly what that is. They clarify in the results, but would be helpful to have that information upfront

**As suggested, we have added a dedicated “Design” subsection immediately preceding the Participants section.**

2. Please provide more information on how the norming ratings on the 1-5 believability scale were used. What were the means? What values were you looking for? Etc.

**We thank the reviewer for raising this point. The 1–5 scale mentioned in the manuscript refers to post‑study engagement checks rather than norming procedures. We have now clarified this in a newly added section titled Participant Engagement. In this section, we report the following: “*Participants completed two brief engagement checks. Most participants reported high motivation (57.7% selected “5” and 32.5% selected “4”), with an overall mean of 4.39 (SD = 0.93). Second, all participants indicated that they took the study seriously (100%). These responses suggest the sample was engaged, supporting confidence in the quality of the self-reported data*.”**

3. Please provide more information on the 17 verbal cues, state what they are, where you got them from, the classifications, who is deciding cues are diagnostic, etc. I went and checked out the original paper, but readers should not have to chase down prior papers to evaluate and understand the study

**As suggested, we have substantially revised the relevant sections of the manuscript to ensure that readers no longer need to consult previous publications to understand the measure.**

- **In the Materials section, we now clearly describe how the 17 verbal cues were selected, specifying their origins in Criteria-Based Content Analysis (CBCA) and Reality Monitoring (RM) frameworks, and detailing how overlapping or less relevant items were excluded following prior literature (e.g., Vrij, 2005; Sporer, 1997).**
- **In the Results section, we explain the basis for our classification of cues as diagnostic or non-diagnostic, following the empirical criteria used in Bogaard et al. (2016) and Bogaard & Meijer (2018), which are based on findings from relevant meta-analyses (e.g., DePaulo et al., 2003; Amado et al., 2015).**
- **Finally, we have included a new Appendix that lists all 17 cues, their original wording (as used in the questionnaire), and their source (CBCA or RM), so that readers can fully understand the instrument and its theoretical basis without referring to earlier studies.**

4. I found the reporting of cue belief results to be a bit harder to follow than expected (p10 starting on line 226). It would be helpful in the results section if the authors could pivot to something like “of the 10 diagnostic cues, they recognized X. Of the 7 nondiagnostic, they false alarmed to X.”

**Thank you for this suggestion. We revised the section as follows: “*As can be seen in Table 1, only 6 out of 17 verbal cues were associated with a significant directional endorsement (p < .001), meaning participants judged liars and truth tellers to differ on these cues. Of these six endorsed cues, only two were diagnostic of deception (i.e., coherence and inconsistencies). When examining participants' broader accuracy in distinguishing diagnostic from nondiagnostic cues, results revealed limited insight: of the 10 diagnostic cues, participants correctly recognized only two; of the seven nondiagnostic cues, five were correctly identified as nondiagnostic, while two were erroneously associated with deception. Furthermore, the response distributions revealed considerable variability in beliefs across individuals, suggesting that there is little shared understanding of which verbal cues are truly diagnostic.*”**

5. For the table, I would swap the * to be indicative of significance as that is more common and then have the bold for it being accurate
   o I would also group them in terms of the “Actual” column such that the “more for liars” are grouped together, then “inconclusive”, then “less for liars” or something to that effect

**We have implemented the first part of the suggestion: statistical significance is now indicated using asterisks (*), and boldface is used to denote accuracy of the endorsed belief. Regarding the second suggestion to reorder the cues, we have chosen to retain the original order as used in Bogaard and Meijer (2018). This allows for a direct comparison with the original study and preserves consistency across replications.**

6. Consider adding a column next to the “Actual” one that has the “Perceived” responses to make this easier to digest for your readers

**As suggested, we added a “perceived” column to Table 1.**

7. Why do the authors think there was only a small effect for true but not deceptive statements? Does the type of cues they were acc/inacc on provide insight? Or the directionality of the cues they were sensitive to? What does that mean for the field/implications, etc. Some elaboration about the implications of these data would strengthen the discussion.

**As suggested, we added two sections to our discussion: one explaining why we found an asymmetric effect and one about possible implications. We included the following:**

(1) ***“The repeated finding that beliefs correlate only with credibility scores of correct statements may be explained by several factors. First, most empirically supported verbal cues signal truthfulness rather than deception (5, 6), meaning accurate cue knowledge provides clearer diagnostic information for truth judgments. Second, individuals with relatively accurate cue knowledge may more readily recognize when statements conform to an expected truthful profile, thereby improving truth detection. In contrast, deceptive statements are more heterogeneous and less consistently marked by the absence of specific cues (3), making them inherently more difficult to identify even with accurate cue knowledge. Third, participants in our study showed poor accuracy in identifying diagnostic cues and frequently endorsed nondiagnostic features as indicative of deception. Consequently, when evaluating lies, they may have relied on invalid or misleading cues, which would limit, or even undermine, any potential benefit of their partial cue knowledge for lie detection.”***
(2) ***“These findings have important implications for deception detection training and practice. Our results showed that cue knowledge showed only modest improvements in truth – but not lie – detection. Consequently, training programs focused simply on increasing cue knowledge will likely bring only limited improvement to lie detection accuracy. Further improvements may come from approaches that go beyond assessing behavioral or verbal cues in isolation. For example, previous research has shown that information-gathering protocols (24) and strategic questioning techniques (25, 26) elicit additional details or create opportunities for checking statements against independent evidence.”***

**Minor points:**

8. The abstract refers to an “original study”. It is clear what that is in the manuscript, but not clear prior to the introduction. I would just name the study in the abstract to clarify this

**We named the study in the abstract.**

9. It would be nice to include some examples of the verbal cues to deception on p4 line 90

**As suggested, we included some examples of used cues: “*Bogaard and Meijer (14) specifically examined whether people’s beliefs about verbal cues such as detail richness, number of contradictions, coherence of statement relate to how they judge the credibility of truthful and deceptive statements.*”**

10. Is there any chance that having the participants provide their cue ratings first could have influenced their performance on the task? Maybe they considered something that they would not have if they had not read about it earlier?

**Thank you for this observation. It is indeed possible that completing the cue belief ratings before the detection task could have primed participants. We have now addressed this in the limitations section: “Third, participants completed the cue belief measure before the lie detection task, which may have primed their attention to specific cues. However, this procedure was consistent with the original study.”**

11. Did the cue belief responses replicate Bogaard and Meijer’s exactly? Any differences? A table for that could be interesting

**We thank the reviewer for this suggestion. We now explicitly compare the current results to Bogaard and Meijer (14) in the Discussion. While we chose not to include a full table due to the high heterogeneity in responses (also observed in the original study), we do report key similarities and differences in the pattern of cue beliefs: *“Regarding cue beliefs, similar to Bogaard and Meijer (14), we found that participants’ beliefs were marked by considerable heterogeneity, reflecting a lack of shared understanding of which verbal cues are truly diagnostic. However, in the original study, participants correctly identified 60% of diagnostic cues (6 out of 10) and 43% of nondiagnostic cues (3 out of 7). In contrast, the current sample showed a more skewed pattern, correctly identifying only 20% of diagnostic cues (2 out of 10), but 71% of nondiagnostic cues (5 out of 7). Thus, although both studies show that laypeople’s cue beliefs are selective and inconsistent, the current findings suggest an even stronger tendency to misidentify valid indicators of deception while being relatively accurate about cues that are actually nondiagnostic. These findings echo prior studies (2, 14, 22, 23) demonstrating that laypeople hold diverse and largely inaccurate beliefs about verbal indicators of deception.”***

**Furthermore, we relate back to the skewedness of our findings to explain why we might have found a lower correlation than the original study: “*This pattern is consistent with the original findings by Bogaard and Meijer (14), who also reported a small but significant correlation between accurate beliefs and credibility judgments of truthful statements (r = .19, p < .004), but no relationship for fabricated statements. However, the effect sizes in the current study were somewhat smaller. This may be due to the more skewed belief patterns observed: participants recognized fewer diagnostic cues correctly than in the original study, which may have limited the potential benefits of cue knowledge for veracity judgments.*”**
